# Bioengineered Plants Can Be a Useful Source of Omega-3 Fatty Acids

**DOI:** 10.1155/2017/7348919

**Published:** 2017-02-21

**Authors:** Waleed Amjad Khan, Hu Chun-Mei, Nadeem Khan, Amjad Iqbal, Shan-Wu Lyu, Farooq Shah

**Affiliations:** ^1^State Key Laboratory of Crop Genetics and Germplasm Enhancement, College of Horticulture, Nanjing Agricultural University, Nanjing, Jiangsu 210095, China; ^2^Laboratory of Biotechnology, Chinese Academy of Tropical Agricultural Sciences, Wenchang, Hainan 571339, China; ^3^Department of Agriculture, Abdul Wali Khan University Mardan, Mardan, Pakistan

## Abstract

Omega-3 fatty acids have proven to be very essential for human health due to their multiple health benefits. These essential fatty acids (EFAs) need to be uptaken through diet because they are unable to be produced by the human body. These are important for skin and hair growth as well as for proper visual, neural, and reproductive functions of the body. These fatty acids are proven to be extremely vital for normal tissue development during pregnancy and infancy. Omega-3 fatty acids can be obtained mainly from two dietary sources: marine and plant oils. Eicosapentaenoic acid (EPA; C20:5 n-3) and docosahexaenoic acid (DHA; C22:6 n-3) are the primary marine-derived omega-3 fatty acids. Marine fishes are high in omega-3 fatty acids, yet high consumption of those fishes will cause a shortage of fish stocks existing naturally in the oceans. An alternative source to achieve the recommended daily intake of EFAs is the demand of today. In this review article, an attempt has, therefore, been made to discuss the importance of omega-3 fatty acids and the recent developments in order to produce these fatty acids by the genetic modifications of the plants.

## 1. Introduction

The fatty acid is an important component of lipids (fat-soluble part of plant, animal, and microorganism cells). Generally, a fatty acid is composed of a straight chain of carbon atoms, with hydrogen atoms (along the chain) at one end and a carboxyl group (-COOH) at the other end. Depending on the nature of the structural chemistry of these chains, fatty acid can be categorized as saturated or unsaturated; the unsaturated fatty acids can be further classified into monounsaturated or polyunsaturated fatty acids.

**Figure 1:**
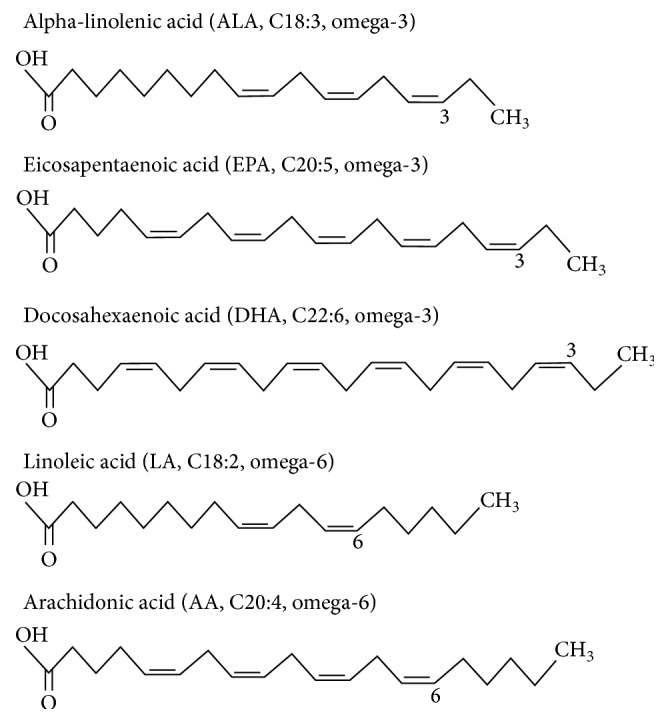
Types of long-chain polyunsaturated fatty acids (LC-PUFAs).

There are a number of fatty acids which do not need to be obtained through diets because they are naturally synthesized by the human body itself and are termed as nonessential fatty acids [1]. On the other hand, omega-3 (*ω*-3) and omega-6 (*ω*-6) fatty acids (Figure 1) are known as essential fatty acids because both of them cannot be synthesized by the body itself and need to be uptaken through diets. The omega-3 fatty acids can be obtained by consuming the plant and marine fish oil. Docosahexaenoic acid (C22:6 n-3) and eicosapentaenoic acid (C20:5 n-3) are primarily obtained through marine resources (mainly fishes and algae). More surprisingly, these compounds are not actually produced by the fishes themselves, yet fishes accumulate them by consuming microalgae or small fishes that have already accumulated them in their tissues [2, 3]. The important forms of omega-3 fatty acids are *α*-linolenic acid (ALA), eicosapentaenoic acid, and docosahexaenoic acid. Likewise, linoleic acid (LA; C18:2 n-6) and arachidonic acid (AA; C20:4 n-6) are the most useful form of omega-6 fatty acids, which can be synthesized by the human body through conversion from linoleic acid.

In the last decade, the polyunsaturated fatty acids (PUFAs) have shown convincing results in the area of biomedical research, especially for their role in prevention against diseases. Omega-3 PUFAs has proved to be critical for human biological systems and are essential for the development of the nervous system during late pregnancy period [4]. Similarly, in adults, its deficiency can lead to severe abnormal conditions like neural and visual disorders, learning disabilities, obesity, cardiovascular disease (CVD), inflammation, and cancer. Omega-3 can be a vital component of the human diet to avoid such abnormal conditions. A number of trial studies have been carried out to investigate the health benefits of the omega-3 PUFAs, including primary and secondary preventions. The aim of the previous studies was not only restricted to their beneficial effects against cardiovascular disease, but also to evaluate its beneficiary roles against inflammatory diseases, Alzheimer's, diabetes, and depression. In this review, the health benefit of omega-3 has been discussed with explanations regarding the alternate potential plant sources for the production of these compounds on the basis of recent studies.

## 2. Imbalance of Omega-6/Omega-3 Fatty Acid Ratio in Modern Diets

The diet habit of human is changed drastically during modern era, when compared with the ancient civilizations [5]. It is assumed that the early foods were rich in omega-3 fatty acids and a well-balanced *ω*-6/*ω*-3 ratio (e.g., 1=1), but the situation is totally changed. Modern diets have a high concentration of saturated fatty acids and also *ω*-6 fatty acids instead of *ω*-3. So, this diet shift has created a disturbance in *ω*-6/*ω*-3 ratio of 1 : 1 to a ratio of higher than 15 : 1 [6]. This conversion of diets has invited a greater risk of cardiovascular diseases and higher susceptibility to other diseases [5, 6]. According to nutrition experts, a diet composed of omega-6/omega-3 in a ratio of less than 5 : 1 is highly recommended [7]. But, in western countries, the diet mainly consumed is richer in omega-6 and estimated as 20 times higher than its omega-3 content [7, 8].

A dose of 0.3–0.5 grams of EPA and 0.8–1.1 grams of DHA is mainly recommended on a daily basis [9]. The European Food Safety Agency (EFSA) has made certain recommendations to intake at least 250 mg/day of combined dose of EPA and DHA for adults to protect them against cardiovascular diseases [10]. Only in a few countries, including Japan, Korea, Philippines, Finland, Norway, Sweden, and Iceland, the population's diet consists of at least 250 mg of *ω*-3 on a daily basis [11]. The statistical data from previous researches show that the current dose in most parts of the world is still far below the required levels recommend by the world health organizations.

## 3. Importance of Omega-3 PUFAs for Human Health

Omega-3 PUFA has now considered being a vital component of human diet after their consistently proven health benefits. It is helpful in maintaining cell physiological processes and other important pathways in the body. It is essential for normal functioning of tissues and organs, and its deficiency can cause abnormalities. Till now, various studies have been conducted to investigate its effects in different health domains, such as cancer, cardiovascular disease, chronic inflammatory diseases, diabetes type 2, neurological disorders, growth and development, depression, and vision [12, 13].

Many studies have been conducted in recent years to investigate and eliminate the causes of modern diseases related to the dietary habits of the humans [14]. Omega-3 PUFAs have provided a breakthrough in medical research, after its presence was noticed in the diet of Greenland Eskimos that have low mortality rate due to coronary heart disease (CHD) [15]. It has become quite clear that if a healthy diet is followed, it can remarkably reduce the risk of CHD in population [16]. For such purpose, ALA, EPA, and DHA are comprehensively studied. Studies have shown the effectiveness of EPA and DHA in primary and secondary prevention of cardiovascular diseases (CVD) [17]. It has been observed through clinical research that the omega-3 PUFA intake lowers the triglycerides (TG) levels in Type-2 Diabetic (T2D) patients [18]. Research studies have proved the effectiveness of marine-based omega-3 PUFAs (EPA and DHA) against some most common types of cancer, including prostate [19], breast [20, 21], and colorectal cancer [22]. The omega-3 PUFAs are also helpful in the formation of protective lipid mediators against inflammatory diseases and disorders [23]. Inflammation may be involved in many chronic diseases such as diabetes, cancer, coronary heart disease, obesity, rheumatoid arthritis, and mental illness [24]. A research which has been made recently also suggested that a low serum DHA is a significant risk factor for the development of Alzheimer's disease [25]. Brain health and other growth and developmental processes that occur throughout the life cycle can be enhanced by taking control diets of balanced n-6/n-3 ratio [26, 27]. Therefore, appropriate amounts of dietary *ω*3 fatty acids are critical for healthy life.

## 4. Present Sources of Omega-3 PUFAs

Plants (especially leafy vegetables and nuts) and deep sea fishes (Tables 1 and 2) are considered to be rich in omega-3 fatty acids and these are currently the main source to obtain alpha-linolenic acid (ALA), eicosapentaenoic acid (EPA), and docosahexaenoic acid (DHA) [28–33].

Alpha-linolenic acid (ALA) is commonly present in plant sources in high concentrations such as walnuts, flax seeds, butternuts, red and black currant seeds, pumpkin seeds, wheat germ, soy and canola oil, and leafy green plants like purslane. Quite recently,* Perilla frutescens* seed oil (PFSO) was also found to be a rich source of omega-3 linolenic acid [34, 35]. Among the plant sources, the highest concentration of alpha-linolenic acid was observed in flax seeds. Furthermore, the best ratio of n-6 : n-3 from a nutritional point of view was found in camelina (1 : 1.4), chia (1 : 3), and hemp seed (1 : 0.4), respectively.

EPA and DHA are the marine-derived sources of omega-3 and mainly found in high concentrations in oily fishes. Fish oil also can be a better source than plant seed oils because of its lower n-6 : n-3 ratio as shown in Table 3 [36]. Salmonidae, Scombridae, and Clupeidae are some oily fish families that contain the highest percentage of EPA and DHA among all fishes. Therefore, Fish oil PUFA has more priority over plant oil PUFA because of its higher concentrations of EPA and DHA. The human body achieves omega-3 from plants in the form of ALA, but they are unable to convert these ALA into essential forms of PUFA (EPA and DHA) because of the lack of such mechanism. So, one can say that the fish oil can be the ideal source to obtain omega-3 PUFA at present.

## 5. Need of Alternative Source for the Production of Omega-3 PUFAs

**Figure 2:**
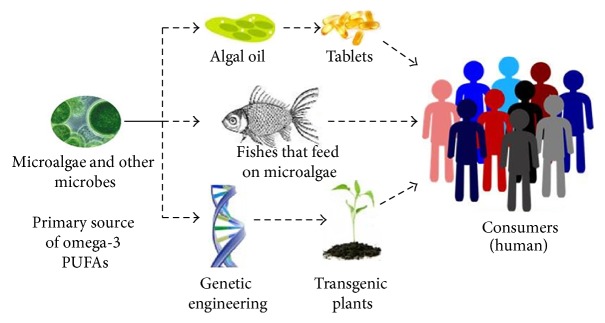
Sustainable sources of omega-3 LC-PUFAs. Microalgae and other microorganisms can serve as a primary source of VLC-PUFAs. The microalgae can be utilized directly or indirectly in the form of algal oil or tablets, respectively. This will also serve as an efficient alternate to the fish (save the fish from extinction) and transgenic plants (quite laborious and expensive to produce).

The demand for omega-3 fatty acids has increased tremendously over the last decade because of its growing beneficial effects in relation to health. However, the average daily intake of EPA and DHA at present is well below the required levels and the world is already facing the lack of sustainable sources to meet the present demands of omega-3 PUFAs. As explained earlier, fishes are currently the primary source of these nutrients, but they are facing problems of shortage due to environmental pollution and human mishandling [37] and may become extinct due to overhunting [38]. The Food and Agriculture Organization has presented a statistical analysis report in 2008, which explained that approximately 53% of the marine fish stocks are utilized by the humans [39]. The individual population of some species of fish were reduced to about 10%, whereas more than hundred species of fish have already became extinct in the oceans [40]. It is estimated that it may take further 40 years to be out of fishery stock, if the mishandling of the fish at this rate continued without any actions [41]. In addition, the fish oils may contain traces of heavy metals such as organic mercury compounds and polychlorinated biphenyls (PCBs) [42]. These concerns have shifted the weight towards adopting new ways of producing LC-PUFAs to cover the required levels through generating genetically modified plants and large-scale production of microalgae [43]. Several attempts have been made to transform oil seed plants into new genetically modified plants that are capable of producing LC-PUFAs [44–46].  Figure 2 shows the efforts for the production of omega-3 PUFAs.

## 6. VLC- PUFA Biosynthetic Pathways

Plant seeds have been used as a direct source of PUFA, but the concentration is very low in higher plants because of the absence of VLC-PUFA biosynthetic pathway to produce EPA and DHA [42]. The conversion of native plant FAs such as LA and ALA to VLC-PUFA requires several FA elongases and desaturases that are not present in higher plants [47]. Considerable efforts have been made to improve the composition of vegetable oil, and tremendous progress has been made in developing the seed VLC-PUFA biosynthetic pathway by producing the required enzymes in plants [42]. The production of EPA and DHA in plants is currently implemented by utilizing algal, bacterial, and yeast genes involved in the PUFA synthesis.

**Figure 3:**
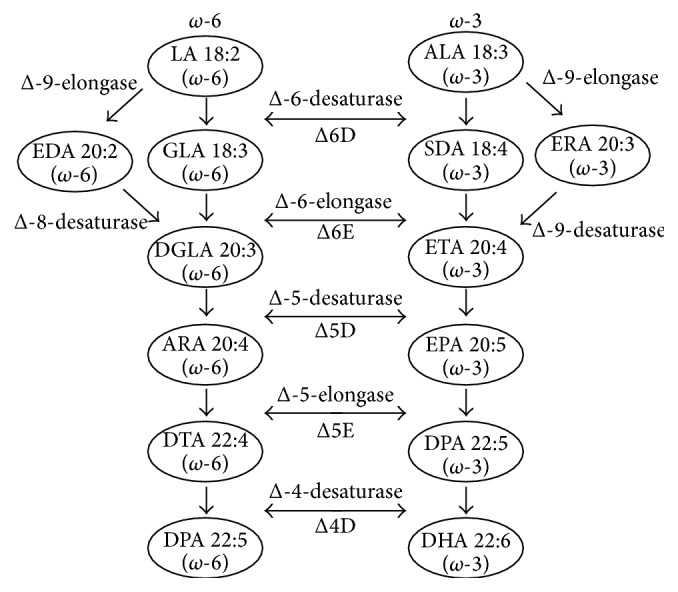
Biosynthesis of the long-chain LC-PUFAs via the conventional pathway [64].

Till now, two pathways have been reported that are responsible for the synthesis of VLC-PUFAs. First pathway is known as aerobic pathway as it involves desaturation and elongation processes, and it needs molecular oxygen in desaturation process. The second pathway known as an anaerobic pathway is widely found in bacteria and several eukaryotic microbes which involve polyketide synthase, which is similar to polyunsaturated fatty acid synthase (PUFA synthase) [48].  Figure 3 shows the conventional biosynthetic pathway for LC-PUFA.

## 7. Microalgal-Based PUFA Production

Use of alternative sources of PUFA oil production from single cell organisms (such as microalgae, bacteria, and yeasts) has gained interest as replacement for fish oil (Table 4). This attention is mainly due to the increasing concerns about global food security and conservation of worldwide fish stocks and also because of high accumulation of toxic substances in fishes [49–51]. Production of long-chain *ω*-3 fatty acids using microalgae is considered to be a promising approach [52]. Various marine microalgal organisms possess the biosynthetic pathway for sequentially alternating desaturation and elongation of C18-PUFA acyl groups, leading to the formation of VLC-PUFAs.

Microalga is mainly consumed by the fishes in marine ecosystems, providing VLC-PUFAs to them by incorporating it into the flesh of their bodies. Microbial cultivation can be a profitable approach for VLC- PUFA production due to high productivity per unit area [53]. Microalgal oil has been extensively reviewed for use in infant formulas as a source of fatty acids [54, 55]. Mutagenesis and other gene modification techniques can assist in improving the PUFA content in these oils. DuPont has developed a yeast strain recently, which has been reported to produce high amounts of EPA levels (55%) [56]. Currently, heterotrophically cultured microalgae are mostly used for DHA production as a food additive (e.g., Martek Biosciences Corporation), but some autotrophic cultivation systems are also under process (e.g., Aurora Algae Inc.). Thraustochytrids (protists) have received more attention in recent years because of their capability to produce high concentrations of omega-3 LC-PUFAs and also they are found abundantly throughout the marine ecosystem [57].

## 8. Approach to Produce Transgenic Plants

Many studies have been done to transfer the omega-3 biosynthesis pathway from microalgae to oilseed plants such as rapeseed and soybean [3]. However, success levels were low and still a lot of bottlenecks exist to solve the problems. The production of omega-3 fatty acids through genetic modification of plants can offer more effectiveness. Production of these oils through microalgae requires higher cost and investments. Therefore, it has been proposed to find the most economical ways to produce cheap, but abundant, sources, which is certainly challenging at present stage. Today, many studies are well focused to make a shift in the metabolic pathway of higher plants to produce fair quantities of omega-3 fatty acids. The synthesis of VLC-PUFAs in plant seeds is an intricate biochemical process, demanding the sequential activity of multiple transgenic enzymes [46]. The first “proof-of concept” was made in* Arabidopsis thaliana* via overexpression of three microbial enzymes (an* Isochrysis galbana* ΔD9 elongase, an* Euglena gracilis* ΔD8 desaturase, and a* Mortierella alpina* D5 desaturase), leading to the formation of ARA and EPA to 6.6% and 3% of the total fatty acids in the leaf tissues [58]. Diversified methods and strategies have been constructed to incorporate the biosynthetic pathway into plants via expression of the desaturase and elongase enzymes, which are the key for different routes to build up EPA and DHA molecules [45]. The predominant sequence (commonly called the “conventional” or “Δ6-pathway”) of enzymatic reaction requires to convert C18 fatty acids to C20 + PUFAs. Such conversions commence with the introduction of a double bond at the Δ6 position, followed by C2 chain elongation and a second desaturation at the Δ5 position in the C20 acyl chain, generating EPA from a-linolenic acid (ALA; 18:3 Δ9,12, 15; n-3) and ARA from linoleic acid (LA; 18:2Δ9,12; n-6) [59].

The entire DHA biosynthetic pathway was later reconstituted in oilseed crop* Brassica juncea* by stepwise metabolic engineering. Transgenic plants produced up to 25% ARA and 15% EPA, as well as up to 1.5% DHA in seeds [46]. Another attempt was also made by introducing four foreign genes in* Arabidopsis* during a study and it resulted in the DHA production of 0.2–0.5% in seed [60]. Recently, some genes Δ6 Des, a Δ6 Elo, and a Δ5 Des obtained from* Marchantia polymorpha* were inserted in tobacco, which has led to the accumulation of 15.5% ARA and 4.9% EPA [61].* Camelina sativa* has proven to be an important source because of the high concentrations of *α*-linolenic acid in the oil from their seeds; since then, it has been widely utilized in aquafeed diets [62, 63]. Transgenic plants (Table 5) can be a huge asset to be a potential source of omega LC-PUFAs, but, in the present circumstances, the resultant amount obtained after plenty of experimental studies was still quite low. So, a better approach to produce high outcomes is highly desirable.

## 9. Conclusions

In recent years, omega-3 LC-PUFAs has appeared to be a valuable asset to human health. The diet based on these fats has shown a great resistance and preventive ability against a wide range of serious health hazards such as cardiovascular disease and reproductive and mental disorders and most importantly against various lethal cancer types.

Obviously, due to its proven significance, its demand level has increased rapidly among the population of the world. Therefore, scientists have been looking for a novel approach to produce these essential, yet beneficial, nutrients in a higher concentration in oilseed plants and vegetables. As we knew, at present, the primary available source for PUFAs is marine fishes, but this source is not sustainable and the fish population was harshly damaged and reduced in recent years due to overconsumption. Transfer of novel genes into higher plants (oilseed) from microalgae can be an effective approach to produce these fatty acids. Many studies have been conducted for such purpose. Some pharmaceutical companies have achieved significant success in attempts to produce EPA and DHA through microbial activities. However, the use of bioengineered plants can be the most promising approach to overcome the global deficits. However, the search of specific enzymes that has the ability to produce healthy oils in appreciable quantities is needed. In current scenario, it has been an important stage for the plant technologists to overcome the demands for LC-PUFAs through adopting a unique but economical approach that can give ground breaking results.

## Figures and Tables

**Table 1:** EPA and DHA content of marine fishes.

Species	Omega-3 (g/kg of fish)			References
	EPA	DHA	Sum	
Sardine (*Sardinops sagax*)	6.6	19.0	25.6	[65]
Herring (*Clupea harengus*)	8.5	8.3	16.8	[65]
Atlantic salmon (*Salmo salar*)	6.2	5.8	12.0	[66]
Surf smelt (*Hypomesus pretiosus*)	3.6	5.7	9.3	[65]
Capelin (*Mallotus villosus*)	3.6	4.6	8.2	[65]
Horse mackerel (*Trachurus trachurus*)	1.6	5.8	7.4	[67]
Red porgy (*Pagrus pagrus*) cultured	2.3	4.0	6.3	[68]
Arctic charr *Salvelinus alpinus*	1.3	2.8	4.1	[69]
Sockeye salmon (*Oncorhynchus nerka*)	0.7	1.9	2.6	[70]
Cod (*Gadus morhua*)	0.6	1.5	2.1	[71]
Red porgy (*Pagrus pagrus*) wild	0.2	1.6	1.8	[68]
Greater weever (*Trachinus draco*)	0.3	1.5	1.8	[68]
Piper gurnard (*Trigla lyra*)	0.3	1.0	1.3	[68]

**Table 2:** Plant sources of polyunsaturated fatty acids [72].

Source (100 g edible portion, raw)	*α*-Linolenic acid in grams
*Nuts and seeds*	
Almonds	0.4
Chia seeds (dried)	3.9
Butternuts (dried)	8.7
Flax seed	22.8
Soybean kernels (roasted or toasted)	1.5
Walnuts, black	3.3
Walnuts, English and Persian	6.8
*Vegetables*	
Beans, navy, sprouted (cooked)	0.3
Broccoli, Cauliflower, Lettuce, Spinach (raw)	0.1
Leeks (freeze-dried)	0.7
Purslane	0.4
Radish seeds, sprouted (raw)	0.7
Soybeans, green (raw)	3.2
Soybeans, mature seeds, fruit	2.1
Avocado (raw, California)	0.1
Raspberry, Strawberry	0.1

**Table 3:** Fatty acid composition (%) of plant oils, fish species, and microalgae.

Oil	SFA	MUFA	PUFA	n3	n6	n6/n3 ratio
Sunflower	12.0	20.5	67.5	0.10	63.2	632
Corn	14.5	29.9	55.6	0.90	50.4	56
Soya	15.6	21.2	63.2	7.30	51.5	7.05
Palm	47.8	37.1	15.1	0.30	10.1	33.66
Olive	14.3	73.0	12.7	0.70	7.8	11.14
Cod liver	22.6	20.7	56.8	19.8	0.9	0.04
Herring	21.3	56.6	22.1	11.9	12	1.01
Salmon	19.9	17.0	63.1	35.3	1.06	0.03
Sardine	30.4	14.5	55.1	28.1	2.2	0.07
Microalgae	37.1	33.8	39.9	32	8.2	0.25

**Table 4:** EPA or/and DHA content of selected microalgal strains.

	Species	Reference
EPA content (g/100 g of FAs)		
29	*Chlorella minutissima*	[73]
26.7	*Nannochloropsis *sp.	[74, 75]
23.4	*Nannochloropsis oceanica*	[76]
~28	*Nannochloropsis salina*	[77]
23.13	*Pinguiococcus pyrenoidosus*	[78]
21.4	*Dunaliella salina*	[79]
DHA content (g/100 g of FAs)		
41	*Crypthecodinium cohnii *sp.	[80]
29.3	*Ceratium horridum*	[81]
EPA + DHA content (g/100 g of FAs)		
36.5 + 23.6	*Phaeodactylum tricornutum*	[82]
41.5	*Pavlova lutheri*	[83]
~28.0	*Isochrysis galbana*	[83]

**Table 5:** Omega-3 contents in transgenic plants.

Oil seed plants	% EPA and/or DHA production	Reference
*Camelina sativa* oil seeds CSTRO, includes model plants	26 EPA 15 EPA 14 DHA	[84] [85]
Transgenic soy bean	20.0 EPA	[86]
*Brassica carinata*	25.0 EPA	[87]
Mustard (BASF)	15.0 EPA 1.5 DHA	[46]

## Abbreviations

PUFA: Polyunsaturated fatty acids
LC: Long-chain
EPA: Eicosapentaenoic acid
DHA: Docosahexaenoic acid.
